# Cardiac function in relation to functional status and fatigue in patients with post-COVID syndrome

**DOI:** 10.1038/s41598-022-24038-3

**Published:** 2022-11-15

**Authors:** Paul Baum, Lisa Do, Lea Deterding, Julia Lier, Ines Kunis, Dorothee Saur, Joseph Classen, Hubert Wirtz, Ulrich Laufs

**Affiliations:** 1 Klinik und Poliklinik für Kardiologie, Universitätsklinikum Leipzig, Liebigstrasse 20, 04103 Leipzig, Germany; 2 Klinik und Poliklinik für Onkologie, Gastroenterologie, Hepatologie, Pneumologie und Infektiologie, Universitätsklinikum Leipzig, Leipzig, Germany; 3 Klinik und Poliklinik für Neurologie, Universitätsklinikum Leipzig, Leipzig, Germany

**Keywords:** Diagnosis, Medical imaging, Cardiology, Health care, Biomarkers, Diagnostic markers

## Abstract

Patients with Post-COVID syndrome (PCS) are frequently referred for cardiologic evaluation. We assessed cardiac function and biomarkers in relation to functional status and fatigue in patients with PCS. This prospective single-center cohort study included 227 patients with persisting symptoms after COVID-19 infection. Most frequent complaints were fatigue (70%), dyspnea (56%), neurocognitive symptoms (34%) and chest pain (28%). Standardized questionnaires were used to assess Post-COVID-Functional-Scale (PCFS) and fatigue (MFI-20). The fatigue severity was inversely related to age and did not correlate with cardiovascular diseases, echocardiographic findings, or biomarkers. Similarly, mild to moderate functional impairment (PCFS 1–3) did not correlate with cardiovascular alterations. However, the subgroup of patients with significant functional impairment (PCFS = 4) had more frequent cardiovascular comorbidities, biomarkers and impaired global longitudinal strain (GLS). Patients with elevated troponin T showed abnormal GLS, reduced left ventricular ejection fraction and impaired tricuspid annular plane systolic excursion. The majority of patients with PCS shows a normal cardiac function. Only the small subgroup of patients with severe functional impairment and patients with elevated troponin T is at risk for impaired cardiac function and likely to benefit from specialized care by a cardiologist.

## Introduction

The post-acute sequelae of SARS-CoV-2 infection (PACS) encompass persistent symptoms beyond four weeks from COVID-19^1,2^. The World Health Organization defines the Post-COVID syndrome (PCS) as symptoms that persist three months after infection, last > two months and are not explained by another disease^3,4^. PACS and PCS are summarized as long-COVID syndrome (LCS). The reported prevalence of PCS differs with the studied population and study design^5–9^. A tendency towards the female sex was observed, but further risk factors remain unknown^10^.

Chronic fatigue is the most frequent symptom among PCS patients^11^. Fatigue is a subjectively perceived exhaustion that follows disproportionately after exertion and does not improve adequately after sleep or rest (post-exertional malaise, PEM). Cardiologic diseases can also cause fatigue and several additional symptoms that are associated with PCS, such as dyspnea, exercise intolerance, palpitations, chest pain, anxiety and depression^11–13^. The risk of cardiovascular events is increased during the acute phase of COVID-19 infection^14^ and there are alterations of cardiovascular function and biomarkers in PACS patients^2,15–17^. However, the effects on ventricular ejection fraction (LVEF), global longitudinal strain (GLS), high sensitive (hs) troponin T and n-terminal pro-brain natriuretic peptide (NT-proBNP) are small and may be primarily observed in individuals with severe courses of COVID-19^14,17^. The association of clinical symptoms with cardiac findings in patients after mild COVID-19 infection referred to cardiology has not been reported. Therefore, this study aimed to assess the correlation of cardiac function including cardiac biomarkers with clinical complaints like fatigue in patients with PCS.

## Methods

This prospective single-center cohort study included 234 patients between April and December 2021 at the outpatient clinic. The inclusion criteria were presentation at least 3 months after the acute course of COVID-19, confirmed COVID-19 infection by SARS-CoV-2 polymerase chain reaction (PCR) test, and at least one persisting symptom 12 weeks after acute course of COVID-19. Individuals below age 18 years were excluded. An initial assessment during the acute course of COVID-19 was not carried out. 2 patients did not have positive PCR and 5 patients withdraw consent, the remaining 227 patients were included and analysed. The clinical course of acute COVID-19 was categorized based on self-reported symptom severity: asymptomatic, mild symptoms (treatment out of hospital), moderate symptoms (treatment in hospital) and severe symptoms (intensive care treatment). All Patients underwent a standardized assessment of functional status, fatigue, depression, anxiety, somatic complaints as well as laboratory testing and echocardiographic examination. All methods were carried out in accordance with current guidelines^18,19^. The study was approved by the local ethical review committee (Ethik-Kommission Leipzig, 431/20-ek), and written informed consent was obtained from all patients.

### Assessment of functional status and fatigue

The five point Post-COVID-Functional-Scale (PCFS) was used to assess the severity of functional impairment and long-term effects of COVID-19 in PCS patients^20^. The scale reaches from 0 (no limitation) to 4 (severe limitation)^21^. Assessment of fatigue severity was performed using the Multidimensional Fatigue Inventory-20 (MFI-20)^22^. The MFI-20 is a self-reporting questionnaire and contains twenty items categorized in five domains with four items in each subscale: general fatigue, mental fatigue, physical activity, motivation and reduced activity. The score in each domain ranges from 4 to 20 points, while higher values indicate more fatigue. The expression of fatigue depends on age and gender^22–24^. The domains were defined positive above the third quartile considering the mean values in the general population^22^. The MFI-20 is validated in healthy individuals and several different disease cohorts^7,23–25^. Additional questionnaires were used to assess depression (Patient Health Questionnaire-9 / PHQ-9), anxiety (Generalized Anxiety Disorder Assessement-7 / GAD-7), somatic complaints Patient Health Questionnaire-15 / PHQ-15)^26–29^.

### Laboratory parameters

Laboratory tests were performed on the day of sample collection at Leipzig University hospital (accredited for ISO 17025 and 15189). Serum hs troponin T and NT-proBNP analyses were performed using the electrochemiluminescence immunoassays “hs troponin T” (REF 09315357190) and “NT-proBNP II” (REF 09315284190) on a cobas® 8000 e801 module, (Roche Diagnostics, Mannheim, Germany). Subgroup analyses were performed in patients with elevated hs troponin T (cut off 14 pg/ml) and elevated NT-proBNP (cut off 125 pg/ml)^30,31^.

To provide information on the immune status the concentration of antibodies against the receptor-binding domain (RBD) of the spike protein and the nucleocapsid protein of SARS-CoV-2 were determined using the commercially available Abbott SARS-CoV-2 IgG II Quant (REF 6R86-22) and SARS-CoV-2 IgG (REF 6S60-22) assays, respectively. Both assays were performed using an ARCHITECT i2000SR system (Abbott, Chicago, USA). To obtain the values for WHO binding antibody units (BAU/ml), the test specific values in arbitrary units (AU/ml) were multiplied by a correction factor of 0.142.

### Echocardiography

Transthoracic echocardiography examinations were performed using GE Vivid E95. Left ventricular (LV) and right ventricular (RV) dimensions, volumes as well as LV and RV function, were measured according to the recommendations of the American Society of Echocardiography (ASE) and the European Association of Cardiovascular Imaging (EACVI)^32^. LV assessment included LV end-diastolic diameter (LVEDD), LV end-diastolic volume (LVEDV), LV end-systolic volume (LVESV), left atrial diameter (LAD), left atrial volume index (LAVI) and left ventricular ejection fraction (LVEF). RV assessment included RV end-diastolic diameter (RVEDD), right atrial area (RAA), tricuspid annular plane systolic excursion (TAPSE) and systolic pulmonary artery pressure (sPAP). LVEF > 50% was defined as preserved LV-function according to current heart failure ESC guidelines^31^. 2-D speckle tracking analysis was performed in the three standard apical planes and global longitudinal strain (GLS) was calculated^33^. As cut-off for GLS we used -16,7% according to the normal ranges for strain measurement^34^.

### Statistical analysis

Statistical analyses were performed using SPSS statistics for windows, version 27.0 Armonk, NY: IBM Corp. Descriptive analyses present continuous data as mean / standard deviation and categorical data as absolute numbers / percentage. Subgroup analyses were performed according to PCFS, MFI-20 as well as LVEF and GLS quartiles. Parameters were tested for normal distribution using Shapiro–Wilk-Test. All parameters without normal distribution were analysed by nonparametric test. Mann–Whitney-U-Test and Kruskal–Wallis-Test were used to test continuous variables. Chi-squared test were used to test categorical variables. A *P* value < 0.05 was considered significant.

## Results

### Patient characteristics

The characteristics of the 227 patients are depicted in Table 1 (PCFS) and Table 2 (MFI-20). 64.3% of the population was female. The mean age was 50 ± 15.1 years. 5.7% of the patients had an asymptomatic, 82.8% a mild, 10.1% a moderate course of COVID 19 with hospitalization, while 1.3% required intensive care. The mean time between infection (date of the first positive PCR) and consultation for PCS was seven months (range: three months to eighteen months). Vaccination became widely available in Germany in the summer 2021. Therefore, only two of the patients in this study had been vaccinated at the time of SARS-CoV2 infection. Approximately 20% of the patients received a vaccination after their SARS-CoV2 infection. No patient had more than two vaccinations. There were no statistical differences between vaccination and any of the parameters assessed. The antibody titer after COVID-19 are shown in Tables 1 and 2.

**Table 1:** Cardiac characteristics and post-COVID-functional-scale (PCFS).

Variables	Overall cohort n = 227	PCFS 0 n = 52	PCFS 1 n = 29	PCFS 2 n = 87	PCFS 3 n = 50	PCFS 4 n = 9	*P*-Value
**Clinical Course of Covid-19**							
Asymptomatic	13 (5.7)	10 (19.2)	0 (0)	2 (2.3)	1 (2.0)	0 (0)	
Mild	188 (82.8)	38 (73.1)	26 (89.7)	78 (89.7)	42 (84)	4 (44.4)	
Moderate (hospital)	23 (10.1)	4 (7.7)	3 (10.3)	7 (8)	7 (14)	2 (22.2)	
Severe (ICU)	3 (1.3)	0 (0)	0 (0)	0 (0)	0 (0)	3 (33.3)	< 0.001*
**Baseline**							
Female [%]	146 (64.3)	31 (59.6)	17 (58.6)	59 (67.8)	34 (68)	5 (55.6)	0.741
Age [y]	48.6 ± 15.1	58.1 ± 13.9	41.9 ± 15.3	44.1 ± 13.8	48.2 ± 11.4	62.2 ± 18.9	< 0.001*
Body Mass Index [kg/m^2^]	27.2 ± 5.4	26.2 ± 4.3	26.2 ± 4.5	27.1 ± 5.7	28.8 ± 5.6	28.1 ± 7.6	0.123
**Clinical presentation**							
NYHA I	99 (43.6)	41 (78.9)	17(58.6)	24 (27.6)	15 (30)	2 (22.2)	
NYHA II	101 (44.5)	10 (19.2)	12 (41.4)	54 (62.1)	22 (44)	3 (33.3)	
NYHA III	27 (11.9)	1 (1.9)	0 (0)	9 (10.3)	13 (26.0)	4 (44.4)	< 0.001*
Systolic blood pressure [mmHg]	142.7 ± 20.1	146.1 ± 19.7	144.0 ± 22.3	141.2 ± 21.3	141.8 ± 17.6	138.8 ± 17.5	0.646
Diastolic heart pressure [mmHg]	85.4 ± 11.2	84.3 ± 11.5	85.6 ± 10.8	86.4 ± 11.8	85.8 ± 10.5	79.7 ± 10.0	0.47
O2-Saturation [%]	97.7 ± 2.3	97.4 ± 3.9	98.0 ± 1.5	98.0 ± 1.5	97.7 ± 1.8	96.3 ± 1.8	0.25
Heart rate [/min]	78.8 ± 14.7	73.6 ± 14.3	82.3 ± 13.6	80.3 ± 14.2	79.6 ± 15.7	78.0 ± 14.0	0.052
**Comorbidities**							
Hypertension	68 (30.1)	16 (30.8)	8 (27.6)	24 (27.6)	15 (30)	5 (55.6)	0.535
Dyslipidaemia	134 (59)	34 (65.4)	12 (41.4)	50 (57.5)	31 (6.2)	7 (77.8)	0.188
Diabetes mellitus	12 (5.3)	4 (7.7)	0 (0)	1 (1.1)	4 (8.2)	3 (33.3)	< 0.001*
Coronary artery disease	6 (2.6)	1 (1.9)	1 (3.4)	1 (1.1)	2 (0.4)	1 (11.1)	0.434
Obesity	60 (27.5)	7 (15.9)	6 (20.7)	22 (25.6)	21 (10.5)	4 (44.4)	0.023*
**Neuropsychiatric assessement**							
PHQ-9 Depression	10.1 ± 4.8	3.9 ± 3.0	7.3 ± 3.5	8.9 ± 4.5	12.2 ± 4.9	13.5 ± 4.6	< 0.001*
GAD-7 Anxiety	7.5 ± 4.7	3.3 ± 3.0	6.1 ± 4.0	7.9 ± 4.5	8.8 ± 5.2	9.4 ± 4.9	< 0.001*
PHQ-15 Somatization	13.7 ± 5.0	6.1 ± 4.4	11.9 ± 4.3	12.7 ± 4.6	16.1 ± 4.9	14.4 ± 4.5	< 0.001*
MFI-20 Fatigue	4.0 ± 1.3	1.3 ± 1.7	3.4 ± 1.6	3.8 ± 1.4	4.2 ± 1.0	4.4 ± 1.4	< 0.001*
**Laboratory**							
Hs troponin T [pg/ml]	5.3 ± 8.3	7.6 ± 7.9	3.8 ± 5.0	3.8 ± 5.1	3.8 ± 4.0	20.7 ± 27.7	< 0.001*
Hs troponin T > 14 pg/ml in [%]	15 (6.6)	3 (5.8)	2 (6.9)	4 (4.7)	1 (2.0)	5 (55)	< 0.001*
NT-proBNP [pg/ml]	98.7 ± 209.2	98.8 ± 87.5	100.9 ± 155.9	68.8 ± 66.0	81.8 ± 95.1	468.6 ± 908.9	< 0.001*
NT-proBNP > 125 pg/ml in [%]	40 (17.7)	13 (25)	4 (13.8)	11 (12.8)	6 (12)	6 (66.7)	< 0.001*
Anti-Nucleocapside [S/CO]	1.7 ± 2.3	1.2 ± 1.6	1.2 ± 1.7	1.8 ± 2.2	1.6 ± 2.2	3.4 ± 4.5	0.189
Anti-RBD [BAU/ml]	1111 ± 1619	1660 ± 1783	1022 ± 1842	1128 ± 1527	1016 ± 1663	457 ± 591	0.447
**Echocardiography**							
*LV-function/dimension*							
LVEF [%]	62.2 ± 5.4	62.9 ± 5.8	62.9 ± 5.0	61.9 ± 5.4	62.1 ± 5.6	60.0 ± 3.5	0.58
GLS [%]	− 19.7 ± 2.2	− 20.6 ± 1.9	− 19.3 ± 1.9	− 19.5 ± 2.3	− 19.4 ± 2.3	− 17.5 ± 1.7	0.001
LAVI [ml/m^2^]	20.7 ± 7.8	22.2 ± 6.8	21.1 ± 8.0	19.4 ± 8.7	20.8 ± 7.2	21.8 ± 5.5	0.411
LVEDV index [ml/m^2^]	52.6 ± 20.9	52.8 ± 10.4	54.1 ± 9.2	54.7 ± 27.7	48.7 ± 12.0	49.5 ± 6.4	0.595
*RV-function/dimension*							
RVEDD [mm]	29.2 ± 4.8	28.9 ± 5.7	29.4 ± 3.7	28.6 ± 4.9	28.6 ± 4.2	35.4 ± 4.7	0.007*
TAPSE [mm]	21.6 ± 3.0	21.8 ± 2.6	22.6 ± 3.0	21.2 ± 2.9	21.4 ± 3.1	20.3 ± 4.7	0.232
sPAP [mmHg]	28.6 ± 8.1	31.1 ± 6.6	27.9 ± 4.8	27.3 ± 5.2	29.3 ± 5.8	38.5 ± 25.0	0.007*
RAA [cm^2^]	12.1 ± 3.6	12.4 ± 4.3	12.7 ± 2.7	11.7 ± 3.8	11.9 ± 3.7	14.1 ± 4.0	0.227
*Diastolic function*							
E/e ‘	7.1 ± 2.1	7.6 ± 1.9	7.1 ± 1.9	6.7 ± 1.8	7.8 ± 2.6	7.3 ± 2.0	0.048*

Continuous variables are presented as mean ± standard deviation, and categorical variables are presented as absolute numbers and percentages.

*PCFS* post COVID functional scale, *ICU* intensive care unit, *NYHA* New York Heart Association, *Obesity* defined as body mass index > 30 kg/m^2^, *O2* oxygen, *PHQ-9* patient health questionnaire-9, *GAD-7* generalized anxiety disorder-7, *PHQ-15* patient health questionnaire-15, *MFI-20* multidimensional fatigue inventory-20, *hs troponin* T high sensitive troponin T, *NT-proBNP* N-terminal pro brain natriuretic peptide, *Anti-RBD* antibody against receptor binding domain / spike protein, *LV* left ventricular, *LVEF* left ventricular ejection fraction, *GLS* global longitudinal strain, *TAPSE* tricuspid annular plain systolic excursion, *LAVI* left atrial volume index, *LVEDV index* left ventricular end diastolic volume indexed, *RVEDD* right ventricular end diastolic diameter, *RAA* right atrial area, *sPAP* systolic pulmonary artery pressure.

**Table 2:** Cardiac characteristics and multidimensional fatigue inventory.

Variables	Overall cohort n = 226	MFI 0/5 n = 32	MFI 1/5 n = 18	MFI 2/5 n = 14	MFI 3/5 n = 27	MFI 4/5 n = 50	MFI 5/5 n = 85	*P-*value
**Clinical Course of Covid-19**								
Asymptomatic	13 (5.8)	7 (21.9)	2 (11.1)	0 (0)	1 (3.7)	0 (0)	3 (3.5)	
Mild	188 (83.2)	23 (71.9)	12 (66.7)	14 (100)	24 (88.9)	45 (90.0)	69 (81.2)	
Moderate (hospitalisation)	23 (10.2)	2 (6.3)	2 (11.1)	0 (0)	2 (7.4)	4 (8.0)	12 (14.1)	
Severe (ICU)	3 (1.3)	0 (0)	1 (5.6)	0 (0)	0 (0)	1 (2.0)	1 (1.2)	0.016*
**Baseline**								
Female [%]	145 (64.2)	20 (62.5)	11 (61.1)	10 (71.4)	16 (59.3)	36 (72)	52 (61.2)	0.794
Age [y]	48.6 ± 15.1	61.5 ± 11.3	60.1 ± 14.4	46.1 ± 10.8	48.3 ± 13.9	41.9 ± 15.5	45.8 ± 13.4	< 0.001*
Body Mass Index [kg/m^2^]	27.2 ± 5.4	26.7 ± 5.2	26.2 ± 3.3	28.3 ± 5.6	28.0 ± 5.2	26.7 ± 5.5	27.4 ± 5.8	0.783
**Clinical presentation**								
NYHA I	98 (43.4)	26 (81.3)	9 (50)	7 (50)	11 (40.7)	15 (30.0)	30 (35.3)	
NYHA II	101 (44.7)	6 (18.8)	8 (44.4)	5 (35.7)	13 (48.1)	26 (52.0)	43 (50.6)	
NYHA III	27 (11.9)	0 (0)	1 (5.6)	2 (14.3)	3 (11.1)	9 (18.0)	12 (14.1)	0.003*
Systolic blood pressure [mmHg]	142.7 ± 20.1	149.3 ± 22.8	146.1 ± 19.5	147.1 ± 17.3	143.4 ± 19.8	140.1 ± 17.3	140.0 ± 21.1	0.229
Diastolic blood pressure [mmHg]	85.4 ± 11.2	85.3 ± 11.4	81.2 ± 10.5	87.2 ± 7.9	87.0 ± 11.7	84.9 ± 10.3	85.8 ± 12.3	0.601
O2-Saturation [%]	97.7 ± 2.3	98.2 ± 1.1	97.8 ± 1.3	97.7 ± 2.1	96.4 ± 5.2	98.0 ± 1.6	97.8 ± 1.6	0.055
Heart rate [/min]	78.8 ± 14.7	75.5 ± 10.8	71.3 ± 13.0	82.9 ± 12.4	75.9 ± 16.9	83.2 ± 15.7	79.2 ± 14.5	0.022
**Cardiovascular Comorbidities**								
Hypertension [%]	68 (30.1)	13 (40.6)	5 (27.8)	5 (35.7)	9 (33.3)	8 (16.0)	28 (34)	0.208
Dyslipidaemia [%]	133 (59)	21 (65.6)	13 (72.2)	7 (50)	16 (59.3)	25 (50)	51 (60)	0.542
Diabetes mellitus [%]	12 (5.3)	2 (6.3)	2 (5.6)	0 (0)	4 (14.8)	1 (2.0)	3 (3.5)	0.130
Coronary artery disease [%]	6 (2.6)	1 (3.1)	0 (0)	0 (0)	2 (7.4)	1 (2.0)	2 (2.4)	0.643
Obesity [%]	59 (27.1)	5 (20)	2 (11.8)	6 (42.8)	10 (37.0)	12 (24)	24 (28.6)	0.147
**Neuropsychiatric assessement**								
PHQ-9 Depression	11.3 ± 4.5	2.8 ± 3.1	4.1 ± 2.4	5.5 ± 1.9	6.8 ± 3.5	8.8 ± 3.7	12.5 ± 4.5	< 0.001*
GAD-7 Anxiety	8.5 ± 4.6	2.4 ± 3.1	3.4 ± 2.1	5.1 ± 2.9	4.6 ± 3.3	5.9 ± 4.6	10.3 ± 3.8	< 0.001*
PHQ-15 Somatization	14.8 ± 4.7	5.1 ± 3.7	7.2 ± 4.0	9.3 ± 3.3	11.1 ± 5.1	12.9 ± 4.0	15.6 ± 4.8	< 0.001*
PCFS Functional Impairment	2.2 ± 0.9	0.2 ± 0.6	1.1 ± 1.3	1.0 ± 1.1	1.8 ± 1.0	2.0 ± 0.9	2.2 ± 1.0	< 0.001*
**Laboratory**								
Hs troponin T [pg/ml]	5.3 ± 8.3	7.6 ± 6.3	6.5 ± 5.0	4.1 ± 3.9	5.8 ± 9.0	5.1 ± 12.8	4.4 ± 6.4	0.526
Hs troponin T > 14 pg/ml in [%]	15 (6.6)	2 (6.3)	1 (5.6)	0 (0)	2 (7.4)	3 (6.0)	7 (8.2)	0.928
NT-proBNP [pg/ml]	98.7 ± 209.2	105.6 ± 76.0	126 ± 119.5	65.3 ± 52.2	77.4 ± 109.6	138 ± 411.9	80.7 ± 94.7	0.685
NT-proBNP > 125 pg/ml in [%]	40 (17.7)	9 (28.1)	7 (38.9)	1 (7.1)	3 (11.1)	9 (18.0)	11 (13.1)	0.051
Anti-Nucleocapside [S/CO]	1.6 ± 2.2	1.5 ± 2.0	1.4 ± 1.5	1.9 ± 2.7	1.6 ± 1.8	1.4 ± 2.0	1.8 ± 2.6	0.186
Anti-RBD [BAU/ml]	1178 ± 1656	1593 ± 1761	1846 ± 1999	1589 ± 1963	1222 ± 1962	824 ± 1107	1025 ± 1612	0.058
**Echocardiography**								
*LV-function/dimension*								
LVEF [%]	62.2 ± 5.4	62.3 ± 5.1	63.6 ± 5.5	62.7 ± 4.9	60.7 ± 5.5	62.7 ± 6.3	62.0 ± 4.9	0.64
GLS [%]	− 19.7 ± 2.2	− 21.2 ± 2.1	− 19.2 ± 2.0	− 19.5 ± 1.8	− 19.8 ± 2.4	− 19.6 ± 2.4	− 19.1 ± 1.9	0.005
LAVI [ml/m^2^]	20.7 ± 7.8	22.6 ± 6.7	21.9 ± 6.9	22.1 ± 7.1	21.2 ± 8.1	19.7 ± 7.9	19.8 ± 8.3	0.550
LVEDV index [ml/m^2^]	54.2 ± 22.6	49.9 ± 9.1	51.5 ± 14.1	53.2 ± 13.2	50.0 ± 12.3	52.3 ± 9.4	55.6 ± 28.4	0.748
*RV-function/dimension*								
TAPSE [mm]	21.6 ± 3.0	21.3 ± 2.3	22.8 ± 3.2	22.3 ± 4.6	21.4 ± 2.7	21.6 ± 3.3	21.2 ± 2.8	0.472
RAA [cm^2^]	11.9 ± 3.7	12.2 ± 4.1	13.2 ± 5.2	12,5 ± 4.1	12.6 ± 2.3	12.3 ± 4.2	11.6 ± 3.4	0.508
RVEDD [mm]	29.1 ± 5.3	28.3 ± 5.3	30.0 ± 5.5	29.9 ± 4.1	29.0 ± 3.6	28.9 ± 5.4	29.3 ± 5.2	0.900
sPAP [mmHg]	29.0 ± 8.6	30.0 ± 5.0	28.9 ± 8.0	28.6 ± 6.1	30.1 ± 8.2	29.9 ± 11.8	28.5 ± 5.8	0.924
*Diastolic function*								
E/e’	7.0 ± 2.0	7.8 ± 1.9	7.3 ± 1.6	7.3 ± 2.0	7.5 ± 2.5	6.9 ± 1.9	7.1 ± 2.1	0.366

Continuous variables are presented as mean ± standard deviation, and categorical variables are presented as absolute numbers and percentages.

*MFI-20* multidimensional Fatigue inventory-20, *ICU* intensive care unit, *NYHA* New York Heart Association, *Obesity* defined as Bbody mass index > 30 kg/m^2^, *O2* oxygen, *PHQ-9* patient health questionnaire-9, *GAD-7* generalized anxiety disorder-7, *PHQ-15* patient health questionnaire-15, *PCFS* post COVID functional scale, *hs troponin* T high sensitive Troponin T, *NT-proBNP* N-terminal pro brain natriuretic peptide, *Anti-RBD* antibody against receptor binding domain/spike protein, *LV* left ventricular, *LVEF* left ventricular ejection fraction, *GLS* global longitudinal strain, *TAPSE* tricuspid annular plain systolic excursion, *LAVI* left atrial volume index, *LVEDV index* left ventricular end diastolic volume indexed, *RVEDD* right ventricular end diastolic diameter, *RAA* right atrial area, *sPAP* systolic pulmonary artery pressure.

**Figure 1:**
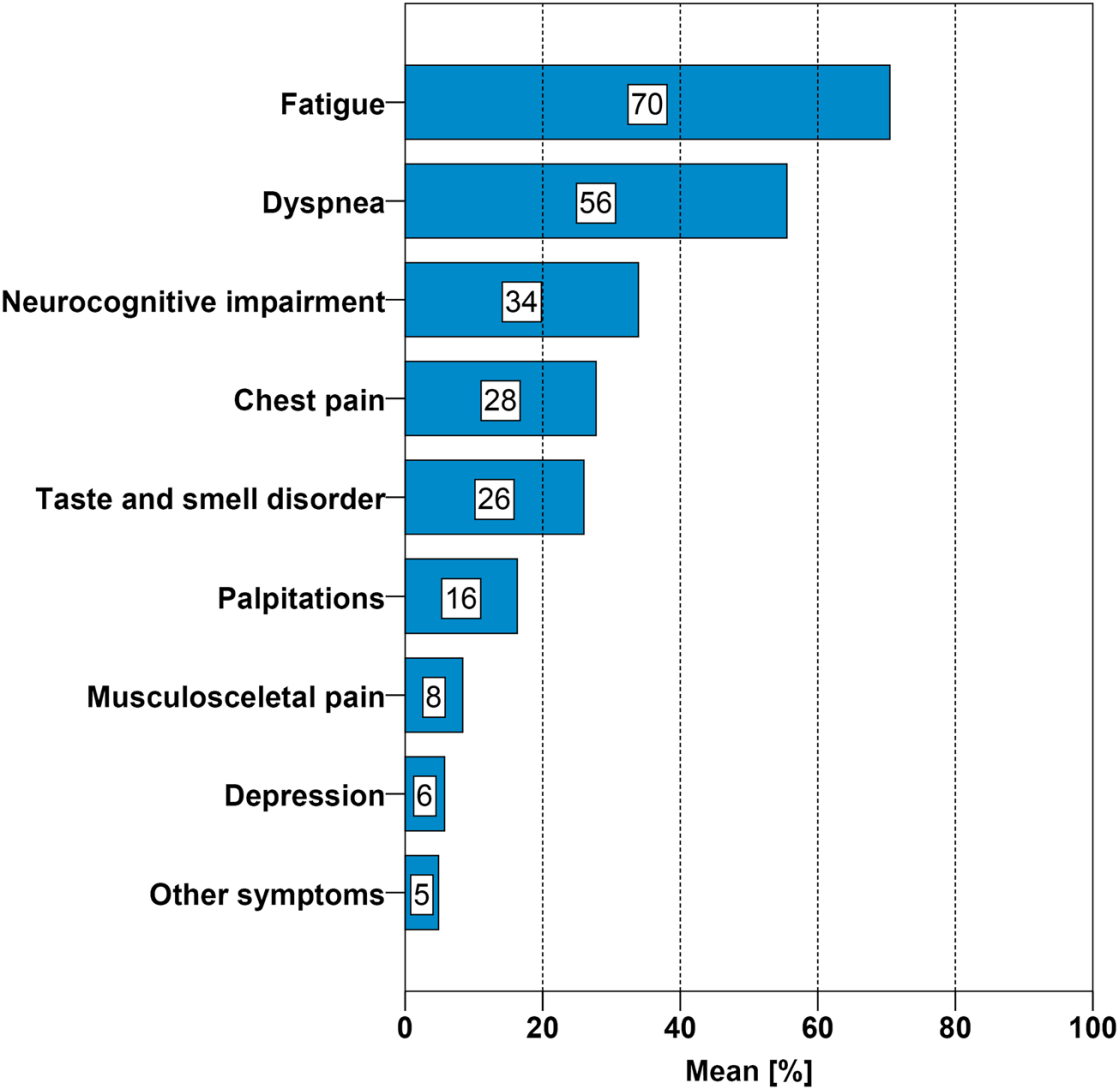
Symptoms at consultation. Prevalence of self-reported symptoms of ambulatory patients with post-COVID-syndrome, n = 227.

The most frequently self-reported symptoms were fatigue (70%) and dyspnea (56%) (Fig. 1). Higher scores in PHQ-9 (depression), GAD-7 (anxiety), and PHQ-15 (somatic symptoms) correlated with functional limitation / PCFS and fatigue / MFI-20 (*P* < 0.001).

### Cardiac characteristics and post-COVID-functional-scale (PCFS)

Patients with higher PCFS scores were more likely to report a severe clinical course of COVID-19 (*P* < 0.001). The functional impairment in PCFS did not correlate with cardiac comorbidities except for diabetes mellitus type 2 and obesity (Table 1). More comorbidities were predictive for higher functional impairment according to PCFS (*P* < 0.013). Patients in the PCFS groups 0 to 3 exhibited laboratory parameters within normal range (Fig. 1). Only 4% of the study population (n = 9) showed significant functional impairment with PCFS category 4. Significantly higher markers of myocardial stress, hs troponin T and NT-proBNP characterized this subgroup. Patients in the group with PCFS 4 had more often diabetes mellitus (*P* < 0.001).

**Figure 2:**
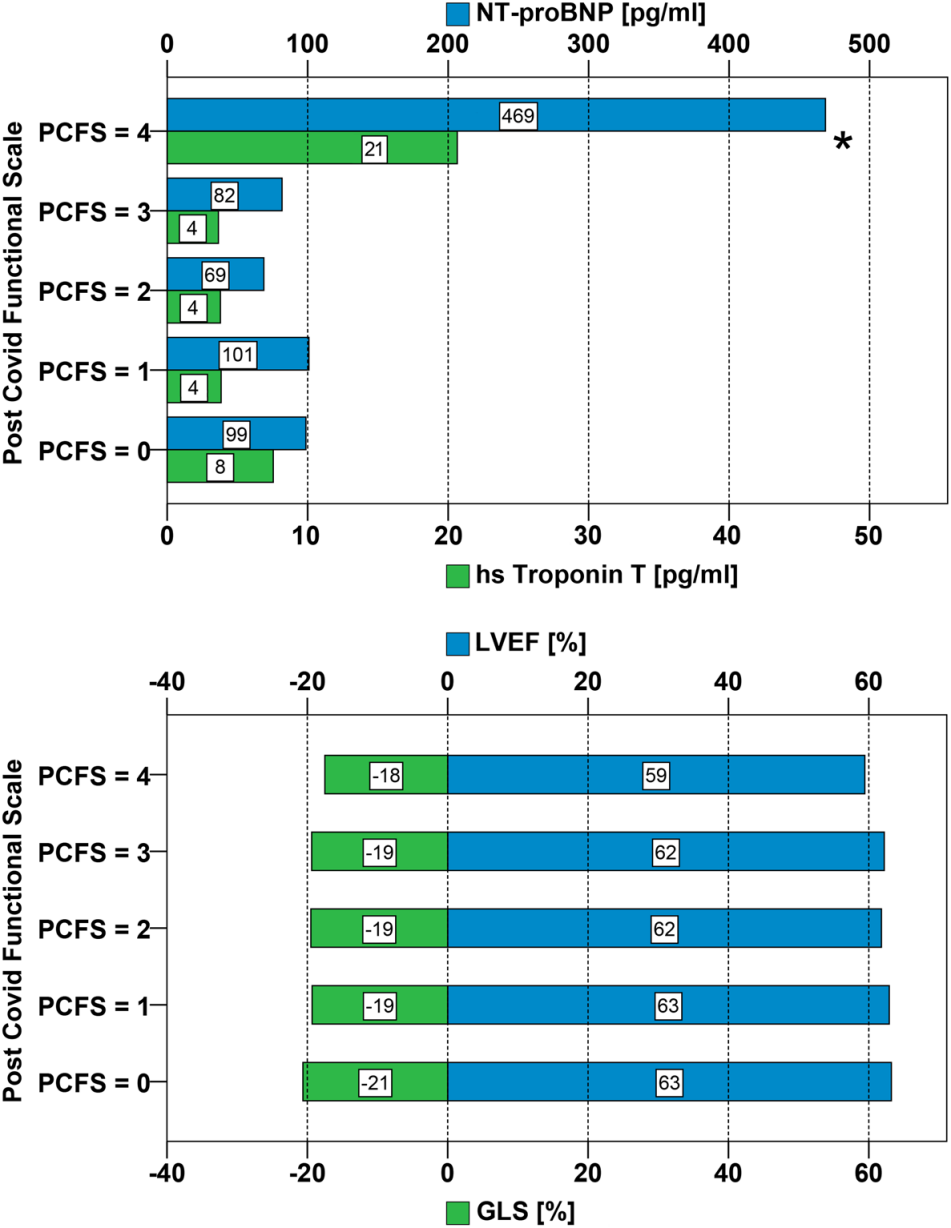
Cardiac biomarkers, echocardiographic parameters and Post-COVID-Functional-Scale. Upper panel: Mean serum concentrations of high sensitive troponin T and N-terminal-pro brain natriuretic peptide stratified by the five point PCFS (*P < 0.001). Lower panel: Mean measurements of LVEF and GLS stratified by PCFS (*P* < 0.001*). PCFS = Post COVID Functional Scale, hs troponin T = high sensitive troponin T; NT-proBNP = N-terminal pro brain natriuretic peptide; LVEF = left ventricular ejection fraction; GLS = global longitudinal strain.

Echocardiography showed no relevant differences for LVEF, TAPSE, LVEDV, LVESV, LAVI, RVEDD, RAA, and sPAP between the PCFS groups. However, patients in PCFS group 4 had higher GLS, RVEDD and sPAP (*P* < 0.001) (Table 1 and Fig. 2) compared to patients of PCFS group 0–3.

### Cardiac characteristics and multidimensional fatigue inventory-20 (MFI-20)

Patients with more positive domains in fatigue were younger (*P* < 0.001). The level of fatigue did not correlate with cardiac comorbidities such as hypertension, dyslipideamia, obesity and diabetes mellitus type 2 in MFI-20 (Table 2). More comorbidities were not predictive for higher scores of the MFI-20 ( *P* = 0.223).

 There was no correlation of positive domains of the MFI-20 with laboratory parameters. Hs troponin T and NT-proBNP were not increased in the highest MFI-20 category (Table 2). The analysed echocardiographic parameters LVEF, TAPSE, LVEDV, LVESV, LAVI, RVEDD, RAA, sPAP showed no significant difference in between the fatigue categories. GLS was marginally higher with -19% in patients with 5/5 pos. domains versus -20% in patients with 0 to 4 pos. domains (*P* = 0.012).

### Characteristics of patients with signs of myocardial pathology

#### LVEF

Patients with lower LVEF were more likely to be male (*P* = 0.004) and have obesity (*P* = 0.013), lower GLS (*P* = 0.001) and lower TAPSE (*P* = 0.004) (supplementary Table 1). Mean values of hs troponin T (*P* = 0.015) as well as the percentage of patients with hs troponin T above the cut off > 14 pg/ml (*P* = 0.003) were higher in the lowest quartile of LVEF.

#### GLS

Patients in the lowest quartile of GLS were more likely to be male (*P* < 0.001), to have obesity (*P* = 0.004) and coronary artery disease (*P* = 0.005). Patients with lower GLS have lower LVEF (*P* = 0.002) and TAPSE (*P* = 0.002) (supplementary Table 2). Hs troponin T and NT-proBNP showed no significant difference concerning GLS quartiles.

#### High sensitive troponin T

**Figure 3:**
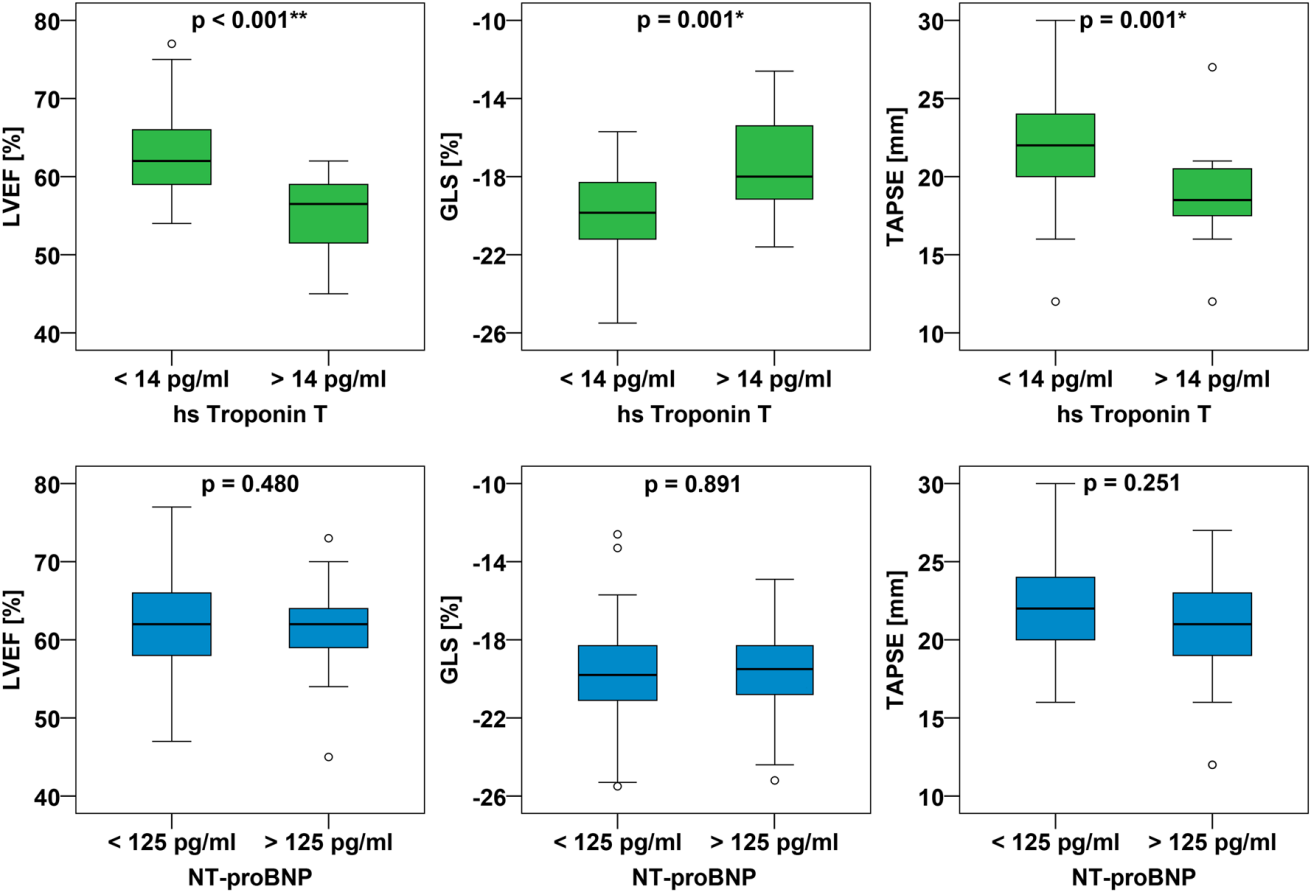
Association between echocardiographic parameters and cardiac biomarkers. *Upper panel* Boxplots of LVEF, GLS, TAPSE according to hs troponin T cut off 14 pg/ml^29^. *Lower panel* Boxplots of LVEF, GLS, TAPSE according to NT-proBNP cut off 125 pg/ml^30^. Hs troponin T = high sensitive troponin T; NT-proBNP = N-terminal pro brain natriuretic peptide; LVEF = left ventricular ejection fraction; GLS = global longitudinal strain; TAPSE = tricuspid annular plain systolic excursion.

15% of the patients showed a hs troponin above 14 pg/ml, 55.6% of these patients had PCFS 4. Patients with hs troponin T levels above 14 pg/ml were older (*P* < 0.001) and more likely to have comorbidities such as hypertension, dyslipidemia, diabetes mellitus, coronary artery disease and obesity (*P* < 0.001). They showed a higher GLS (*P* = 0.008), sPAP (*P* = 0.01), LVEDV (*P* = 0.04) as well as a lower LVEF, (*P* < 0.001) and TAPSE (*P* = 0.001) (Fig. 3). Of note, none of the patients with normal hs troponin T had impaired LVEF or GLS.

#### N-terminal pro-brain natriuretic peptide

40% of the patients had an NT-proBNP above 125 pg/ml, 15% of these patients had PCFS 4. Patients with NT-proBNP serum concentrations > 125 pg/ml were more likely to have hypertension (*P* = 0.003), diabetes mellitus (*P* = 0.042) and coronary artery disease (*P* = 0.01). Echocardiographic quantifications of GLS, LVEF or TAPSE were not different in individuals with NT-proBNP > 125 pg/ml (Fig. 3).

## Discussion

The appropriate approach to the cardiologic evaluation of the large population of patients with post COVID-19 syndrome remains challenging since the reported symptoms, specifically fatigue, dyspnea and chest pain, can be caused by cardiac, pulmonary, muscular and neuropsychiatric diseases^35–37^. This prospective study provides a quantitative characterization of the functional status (PCFS) and fatigue (MFI-20) in relation to cardiac biomarkers and detailed echocardiographic assessment of a typical PCS patient population presenting for cardiologic examination. The main finding of the study is that the majority of PCS patients show cardiac biomarkers and echocardiographic parameters within normal limits despite symptoms of fatigue, dyspnea or chest pain. Importantly, normal hs troponin T levels virtually ruled out abnormal cardiac function in this population. Only a small subgroup of patients (< 5%) showed signs of impaired cardiac function. These patients can be identified by severe functional impairment and / or elevated hs troponin T.

The heterogenous symptoms of the PCS widely overlap with symptoms of cardiovascular disease^12^. Due to the large and growing prevalence of PCS, the challenge for the consulted cardiologist is to allocate the optimal diagnostic work-up avoiding both over- and under-testing. The difficulty is the discrepancy between measurable organ dysfunction and subjectively perceived symptoms in PCS^38^. Our data show that symptoms related to an impaired functional status but not symptoms related to fatigue predict cardiac pathologies. Persisting high scores of PCFS have been observed after initial hospitalization and hypoxemia inducing pneumonia^39–41^. Longitudinal studies of PCS show that an impaired functional status reduces the quality of life regardless to severity of COVID-19^42–45^. In our study, patients with the highest functional impairment (PCFS grade 4) showed evidence for cardiac involvement with elevated cardiac biomarkers and abnormal echocardiographic findings consistent with literature^14,46^. The most likely explanation for the cardiac pathologies are pre-existing cardiac diseases and effects caused by the significantly more severe acute phase of COVID-19 in this subgroup. This small and easily identifiable subgroup of patients are therefore likely to benefit from the specialized care of a cardiologist.

Our study confirms consistent with many observational studies that fatigue is the most common symptom in patients with PCS^1,6,10^. The pathogenesis of fatigue in PCS is multifactorial. A post viral and immunological etiology for chronic fatigue syndrome (CFS) has been proposed for several viral disease including coxsackie, ebstein barr, influenza and varicella virus^47^. However, there is a gap in laboratory and imaging diagnostic in CFS patients. Thus, CFS remains a clinical diagnosis^48^. To date there is no evidence that fatigue is caused by cardiovascular sequelae of COVID-19^49^. As confirmed in our study, the incidence of neuropsychiatric disease including depression, anxiety disorder, and somatization in PCS is high and may explain neurocognitive symptoms like fatigue^50,51^. Therefore a systematical screening for CFS should be performed and neuropsychiatric differential diagnosis should be considered in PCS patients. Our study confirms the importance of fatigues for the symptoms reported by patients with PCS, however, fatigue does not appear to be associated with cardiac pathologies and per se does not require cardiology examinations.

Cardiovascular sequalae, defined as acute or subacute perimyocarditis, acute coronary syndrome, arrhythmias and pulmonary embolisms are not limited to the acute course of COVID-19^9,52^. Hs troponins and natriuretic peptides are sensitive biomarkers of cardiomyocyte damage and cardiac wall stress, respectively. Higher concentrations of troponin and NT-proBNP were also found in PCS patients after mild to moderate infection of SARS-COV2 compared with matched healthy controls^17^. In COVID-19 patients, higher concentrations of hs troponin T are potent markers for cardiac injury and predictive for higher mortality^53^. As a result with practical importance, our study shows that hs troponin is valuable to distinguish between PCS patients with and without impaired cardiac function. Hs troponin T may be used to detect cardiac involvement and simplifies the decision to refer a PCS patient to a cardiologist.

Several cohort studies have characterized echocardiographic findings during acute and post-acute SARS-COV2 infection^16,54,55^. Speckle tracking deformation abnormalities, especially reduced GLS are highly prevalent in the acute phase of the infection^56,57^. Lower GLS are also evident in PCS patients compared to healthy controls^58^. As an additional observation, our study shows an association between GLS and obesity, higher age and high functional impairment. Abnormal global longitudinal strain is also associated with impaired prognosis^56^. These data support speckle tracking analysis as a sensitive tool to detect cardiac damage in PCS patients that may be missed by the assessment of the left ventricular ejection fraction alone.

## Limitations

This is a single-center study and the majority of the population was caucasian. The data need to be confirmed in other ethnicities and health care systems. The median observational period was seven months. Therefore, very long-term effects cannot be excluded. Effects may be under- or overestimated due to convalescence or potential disease aggravation of PCS. We do not have a healthy control group, therefore non-viral effects of the pandemic are difficult to quantitate. The data set the stage and provide the baseline for a long-time follow-up in PCS that cannot be available at this time and for important comparison with other viral infections, e.g. influenza.

## Conclusion

The study shows that the majority (> 95%) of patients with PCS after COVID-19 infection has normal cardiac function assessed by echocardiography without any discernable cardiovascular disease—despite symptoms of fatigue, dyspnea or chest pain. The distinct subgroups of patients with a history of complicated COVID-19 infection requiring hospitalization, patients with severe functional impairment, a higher number of comorbidities and patients with elevated high sensitivity troponin are at risk for impaired cardiac function and are therefore likely to benefit from specialized examination and treatment by a cardiologist.

## Supplementary Information

Supplementary Information.

## Data Availability

The datasets generated during and/or analysed during the current study are available from the corresponding author on reasonable request.
