# Supplementary material for: Cardiac function in relation to functional status and fatigue in patients with post-COVID syndrome
Source: Sci Rep. 2022 Nov 15;12:19575. doi: 10.1038/s41598-022-24038-3 (PMC9664421; doi:10.1038/s41598-022-24038-3)
Supplement: Supplementary file 1 — Supplementary Information. [file 41598_2022_24038_MOESM1_ESM.docx]

| **Variables** | **Overall cohort**  **n=183** | **LVEF > 66%**  **n=45** | **LVEF 66-62%**  **n=48** | **LVEF 62-58%**  **n=45** | **LVEF < 58%**  **n=45** | ***p*-Value** |
| --- | --- | --- | --- | --- | --- | --- |
| **Clinical Course of Covid-19** |  |  |  |  |  |  |
| asymptomatic | 10 (5.5) | 3 (6.7) | 4 (8.3) | 2 (4.4) | 1 (2.2) |  |
| mild | 151 (82.5) | 39 (86.7) | 38 (79.2) | 37 (82.2) | 37 (82.2) |  |
| moderate (hospitalization) | 19 (10.4) | 3 (6.7) | 6 (12.5) | 5 (11.1) | 5 (11.1) |  |
| severe (ICU) | 3 (1.6) | 0 (0) | 0 (0) | 1 (2.2) | 2 (4.4) | 0.68 |
| **Baseline** |  |  |  |  |  |  |
| Female [%] | 20 (44.4) | 36 (80) | 59 (67.8) | 33 (68.8) | 30 (66.7) | 0.004* |
| Age [y] | 48.5 ± 15.7 | 44.4 ± 15.7 | 50.0 ± 16.0 | 49.1 ± 15.1 | 49.8 ± 15.9 | 0.361 |
| Body Mass Index [kg/m²] | 27.1 ± 5.5 | 25.1 ± 4.5 | 26.7 ± 5.4 | 27.9 ± 6.2 | 27.9 ± 6.2 | 0.002* |
| **Clinical presentation** |  |  |  |  |  |  |
| NYHA I | 76 (41.5) | 22 (48.9) | 25 (52.1) | 15 (33.3) | 14 (31.1) |  |
| NYHA II | 82 (44.8) | 19 (42.2) | 19 (39.6) | 23 (51.1) | 21 (46.7) |  |
| NYHA III | 25 (13.7) | 4 (8.9) | 4 (8.3) | 7 (15.6) | 10 (22.2) | 0.182 |
| Systolic blood pressure [mmHg] | 142.4 ± 19.7 | 135.8 ± 13.1 | 148.4 ± 22.1 | 136.5 ± 16.5 | 148.1 ± 22.2 | 0.001* |
| Diastolic blood pressure [mmHg] | 84.7 ± 10.8 | 83.9 ± 9.0 | 84.2 ± 11.2 | 83.9 ± 10.3 | 86.9 ± 12.5 | 0.558 |
| O2-Saturation [%] | 97 ± 2.5 | 98.3 ± 1.3 | 97.9 ± 1.9 | 97.8 ± 1.7 | 97.1 ± 4.1 | 0.165 |
| Heart rate [/min] | 78.5 ± 14.9 | 81.9 ± 15.5 | 76.3 ± 14.3 | 79.1 ± 12.9 | 77.3 ± 16.3 | 0.319 |
| **Comorbidities** |  |  |  |  |  |  |
| Hypertension [%] | 55 (30.2) | 6 (13.3) | 17 (36.2) | 16 (35.6) | 16 (35.6) | 0.044* |
| Dyslipidaemia [%] | 119 (65) | 24 (53.3) | 34 (70.8) | 30 (66.7) | 31 (68.9) | 0.288 |
| Diabetes mellitus [%] | 11 (6) | 1 (2.2) | 2 (4.4) | 2 (4.4) | 6 (13.3) | 0.117 |
| Coronary artery disease [%] | 5 (2.7) | 0 (0) | 0 (0) | 2 (4.4) | 3 (6.7) | 0.126 |
| Obesity [%] | 48 (26.4) | 6 (13.4) | 9 (18.8) | 17 (38.6) | 16 (35.6) | 0.013* |
| **Neurocognitive assesement** |  |  |  |  |  |  |
| PHQ-9 Depression | 8.4 ± 5.1 | 8.4 ± 5.4 | 8.5 ± 5.3 | 8.3 ± 4.8 | 7.8 ± 5.2 | 0.943 |
| GAD-7 Anxiety | 6.5 ± 4.7 | 6.6 ± 4.5 | 6.6 ± 5.0 | 6.3 ± 4.6 | 6.1 ± 4.8 | 0.958 |
| PHQ-15 Somatization | 11.8 ± 5.7 | 11.0 ± 5.8 | 11.7 ± 5.5 | 12.5 ± 5.8 | 11.2 ± 6.0 | 0.612 |
| MFI -20 Fatigue | 3.2 ± 1.8 | 3.2 ± 1.9 | 3.0 ± 2.0 | 3.6 ± 1.6 | 3.1 ± 1.9 | 0.728 |
| PCFS Functional Impairment | 1.7 ± 1.2 | 1.5 ± 1.1 | 1.5 ± 1.3 | 2.0 ± 1.2 | 1.6 ± 1.2 | 0.715 |
| **Laboratory Testing** |  |  |  |  |  |  |
| Hs troponin T [pg/ml] | 5.4 ± 8.6 | 3.0 ± 2.7 | 5.2 ± 5.6 | 5.1 ± 7.6 | 8.4 ± 13.9 | 0.015* |
| Hs troponin T > 14 pg/ml in [%] | 12 (6.6) | 0 (0) | 1 (2.1) | 3 (6.8) | 8 (17.8) | 0.003* |
| NT-proBNP [pg/ml] | 100.3 ± 225.5 | 67.6 ± 49.3 | 93.8 ± 74.3 | 93.1 ± 123.1 | 146.5 ± 427.9 | 0.344 |
| NT-proBNP > 125 pg/ml in [%] | 33 (18.1) | 5 (11.1) | 12 (25) | 8 (18.2) | 8 (17.8) | 0.388 |
| Anti-Nucleocapside [S/CO] | 1.7 ± 2.3 | 1.4 ± 2.2 | 1.8 ± 2.8 | 2.2 ± 2.2 | 1.3 ± 1.9 | 0.115 |
| Anti-RBD [BAU/ml] | 1219 ± 1626 | 633 ± 1035 | 1236 ± 1487 | 1506 ± 1812 | 1534 ± 2100 | 0.085 |
| **Echocardiographic parameters** |  |  |  |  |  |  |
| *LV - function / dimension* |  |  |  |  |  |  |
| GLS [%] | -19.7 ± 2.2 | -20.6 ± 2.2 | -19.9 ± 1.8 | -19.6 ± 2.3 | -18.5 ± 2.1 | 0.001* |
| LVEDV index [ml/m²] | 52.7 ± 18.6 | 54.4 ± 13.1 | 57.0 ± 30.4 | 48.5 ± 8.8 | 50.5 ± 12.3 | 0.03* |
| LAVI [ml/m²] | 20.8 ± 7.8 | 20.6 ± 7.9 | 21.0 ± 8.2 | 20.4 ± 6.9 | 20.6 ± 8 | 1.0 |
| *RV - function / dimension* |  |  |  |  |  |  |
| RVEDD [mm] | 29.1 ± 5 | 27.5 ± 4.9 | 30.3 ± 4.9 | 29.0 ± 4.0 | 29.1 ± 5.8 | 0.73 |
| RAA [cm²] | 12.3 ± 3.8 | 11.6 ± 4.2 | 12.3 ± 3.7 | 12.2 ± 3.0 | 12.5 ± 4.1 | 0.309 |
| TAPSE [mm] | 21.6 ± 3 | 22. ± 3.0 | 22.1 ± 2.9 | 21.6 ± 3.0 | 20.3 ± 2.8 | 0.004* |
| sPAP [mmHg] | 29.2 ± 7.9 | 28.6 ± 4.8 | 29.2 ± 6.4 | 28.3 ± 6.6 | 30.8 ± 12.2 | 0.826 |
| *Diastolic function* |  |  |  |  |  |  |
| E/e' | 7.2 ± 2.1 | 7.0 ± 1.7 | 7.3 ± 1.7 | 6.9 ± 2.2 | 7.6 ± 2.5 | 0.291 |

**Supplementary table 1**

| **Variables** | **Overall cohort**  **n=171** | **GLS <-21%**  **n=43** | **GLS -21% to -19.8%**  **n=42** | **GLS -19.8% to -18.2%**  **n=43** | **GLS > -18.2%**  **n=43** | ***p*-Value** |
| --- | --- | --- | --- | --- | --- | --- |
| **Clinical Course of Covid-19** |  |  |  |  |  |  |
| asymptomatic | 10 (5.8) | 4 (9.3) | 2 (5.4) | 3 (7) | 1 (2.3) |  |
| mild | 142 (83.0) | 37 (86.0) | 34 (81) | 37 (86) | 34 (79.1) |  |
| moderate (hospital) | 16 (9.4) | 2(4.7) | 6 (14.3) | 2 (4.7) | 6 (14) |  |
| severe (ICU) | 3 (1.8) | 0 (0) | 0 (0) | 1 (2.3) | 2(4.7) | 0.359 |
| **Baseline** |  |  |  |  |  |  |
| Female [%] | 111 (64.9) | 36 (83.7) | 28 (75.7) | 37 (63.8) | 10 (30.3) | <0.001* |
| Age [y] | 48.3 ± 15.9 | 49.3 ± 15.4 | 48.4 ± 15.2 | 44.7 ± 17.9 | 50.7 ± 15.1 | 0.578 |
| Body Mass Index [kg/m²] | 26.8 ± 5.3 | 25.2 ± 3.6 | 26.9 ± 4.8 | 26.7 ± 6.0 | 28.6 ± 6.0 | 0.015* |
| **Clinical presentation** |  |  |  |  |  |  |
| NYHA I | 73 (42.7) | 22 (51.2) | 19 (45.2) | 18 (41.9) | 14 (32.6) |  |
| NYHA II | 76 (44.4) | 16 (37.2) | 20 (47.6) | 21 (48.8) | 19 (44.2) |  |
| NYHA III | 22 (12.9) | 5 (11.6) | 3 (7.1) | 4 (9.3) | 10 (23.3) | 0.255 |
| Systolic blood pressure [mmHg] | 141.4 ± 18.8 | 137.7 ± 18.1 | 143.9 ± 20.4 | 140.7 ± 17.4 | 143.9 ± 19.6 | 0.453 |
| Diastolic blood pressure [mmHg] | 84.4 ± 10.7 | 81.3 ± 9.8 | 83.1 ± 9.3 | 86.4 ± 12.0 | 87.1 ± 11.0 | 0.032* |
| O2-Saturation [%] | 97.8 ± 2.6 | 98.3 ± 1.2 | 98.1 ± 1.5 | 97.1 ± 4.4 | 97.5 ± 1.6 | 0.101 |
| Heart rate [/min] | 78.8 ± 15.0 | 77.6 ± 14.5 | 77.5 ± 12.8 | 80.2 ± 18.5 | 79.9 ± 13.6 | 0.463 |
| **Comorbidities** |  |  |  |  |  |  |
| Hypertension [%] | 50 (29.4) | 7 (16.3) | 15 (35.7) | 10 (23.8) | 18 (41.2) | 0.042* |
| Dyslipidaemia [%] | 110 (64.3) | 28 (65.1) | 28 (66.7) | 25 (58.1) | 29 (67.4) | 0.799 |
| Diabetes mellitus [%] | 9 (5.3) | 2 (4.7) | 2 (4.8) | 2 (4.7) | 3 (7) | 0.953 |
| Coronary artery disease [%] | 3 (1.8) | 0 (0) | 0 (0) | 0 (0) | 3 (7.1) | 0.028* |
| Obesity [%] | 42 (24.7) | 5 (11.6) | 7 (16.7) | 12 (27.9) | 18 (42.9) | 0.004* |
| **Neurocognitive assesement** |  |  |  |  |  |  |
| PHQ-9 Depression | 8.0 ± 5.3 | 7.1 ± 5,4 | 9.7 ± 5.5 | 7.0 ± 4.6 | 8.8 ± 4.7 | 0.070 |
| GAD-7 Anxiety | 6.1 ± 4.7 | 5.4 ± 4.8 | 7.2 ± 4.6 | 5.8 ± 4.6 | 7.0 ± 4.8 | 0.213 |
| PHQ-15 Somatization | 11.3 ± 5.9 | 10.6 ± 5.9 | 12.8 ± 6.0 | 10.6 ± 5.6 | 12.1 ± 5.5 | 0.492 |
| MFI-20 Fatigue | 3.1 ± 1.9 | 2.6 ± 2.0 | 3.8 ± 1.7 | 2.9 ± 1.9 | 2.9 ± 1.9 | 0.115 |
| PCFS Functional Impairment | 1.5 ± 1.2 | 1.3 ± 1.2 | 1.7 ± 1.3 | 1.4 ± 1.1 | 2.0 ± 1.1 | 0.074 |
| **Laboratory** |  |  |  |  |  |  |
| Hs troponin T [pg/ml] | 5.4 ± 8.8 | 4.7 ± 7.3 | 4.7 ± 5.9 | 6.0 ± 13.8 | 6.2 ± 6.1 | 0.556 |
| Hs troponin T > 14 pg/ml in [%] | 11 (6.5) | 1 (2.3) | 1 (2.4) | 3 (7) | 6 (14) | 0.97 |
| NT-proBNP [pg/ml] | 97.8 ± 228.7 | 87.1 ± 83.3 | 85.7 ± 78.1 | 142.9 ± 432.2 | 73.6 ± 91.1 | 0.143 |
| NT-proBNP > 125 pg/ml in [%] | 30 (17.6) | 7 (23.3) | 7 (23.3) | 9 (30) | 7 (23.3) | 0.872 |
| Anti-Nucleocapside [S/CO] | 1.7 ± 2.3 | 1.2 ± 1.4 | 1.3 ± 1.9 | 1.5 ± 1.8 | 2.9 ± 3.2 | 0.061 |
| Anti-RBD [BAU/ml] | 1210 ± 1657 | 1444 ± 1907 | 1394 ± 1774 | 899 ± 1478 | 1060 ± 1375 | 0.470 |
| **Echocardiographic parameters** |  |  |  |  |  |  |
| *LV - function / dimension* |  |  |  |  |  |  |
| LVEF [%] | 62.3 ± 5.4 | 64.2 ± 5.0 | 63.0 ± 5.0 | 62.0 ± 5.6 | 60.1 ± 5.4 | 0.002* |
| LVEDV index [ml/m²] | 53.3 ± 18.9 | 53.5 ± 11.1 | 53.0 ± 8.7 | 54.6 ± 32.5 | 51.8 ± 14.0 | 0.865 |
| LAVI [ml/m²] | 20.9 ± 7.8 | 22.6 ± 7.5 | 21.4 ± 7.2 | 19.5 ± 8.5 | 19.6 ± 7.3 | 0.188 |
| *RV - function / dimension* |  |  |  |  |  |  |
| RVEDD [mm] | 29.0 ± 5.0 | 28.8 ± 3.9 | 28.9 ± 5.4 | 28.6 ± 5.8 | 29.3 ± 4.6 | 0.810 |
| RAA [cm²] | 12.2 ± 3.8 | 12.5 ± 4.1 | 12.3 ± 3.7 | 11.6 ± 3.9 | 12.1 ± 3.2 | 0.558 |
| TAPSE [mm] | 21.6 ± 3.0 | 22.6 ± 2.3 | 22. 2 ± 2.7 | 21.0 ± 3.1 | 20.4 ± 3.3 | 0.002* |
| sPAP [mmHg] | 29.3 ± 7.9 | 30.5 ± 5.8 | 29.6 ± 6.5 | 29.1 ± 11.7 | 27.8 ± 6.2 | 0.810 |
| *Diastolic function* |  |  |  |  |  |  |
| e/E‘ | 7.1 ± 2.0 | 7.1 ± 1.9 | 7.1 ± 1.7 | 7.0 ± 1.7 | 7.3 ± 2.4 | 0.952 |

**Supplementary table 2**

**Supplementary tables**

**Supplementary table 1 Characteristics of patients according to left ventricular ejection fraction (LVEF)**

Continuous variables are presented as mean ± standard deviation, and categorical variables are presented as absolute numbers and percentages.

LVEF = left ventricular ejection fraction; ICU = intensive care unit; NYHA = New York Heart Association; Obesity = defined as Bbody mass index > 30kg/m2; O2 = oxygen, PHQ-9 = Patient Health Questionnaire -9; GAD-7 = Generalized Anxiety Disorder-7; PHQ-15 = Patient Health Questionnaire-15; MFI-20 = Multidimensional Fatigue Inventory-20; PCFS = Post COVID Functional Scale; hs troponin T = high sensitive Troponin T; NT-proBNP = N-terminal pro brain natriuretic peptide; Anti-RBD = antibody against receptor binding domain / spike protein; LV = left ventricular; LVEF = left ventricular ejection fraction; GLS = global longitudinal strain; TAPSE = tricuspid annular plain systolic excursion; LAVI = left atrial volume index; LVEDV index = left ventricular end diastolic volume indexed; RVEDD = right ventricular end diastolic diameter; RAA = right atrial area; sPAP = systolic pulmonary artery pressure.

**Supplementary table 2 Characteristics of patients according to global longitudinal strain (GLS)**

Continuous variables are presented as mean ± standard deviation, and categorical variables are presented as absolute numbers and percentages.

GLS = global longitudinal strain; ICU = intensive care unit; NYHA = New York Heart Association; Obesity = defined as Bbody mass index > 30kg/m2; O2 = oxygen, PHQ-9 = Patient Health Questionnaire -9; GAD-7 = Generalized Anxiety Disorder-7; PHQ-15 = Patient Health Questionnaire-15; MFI-20 = Multidimensional Fatigue Inventory-20; PCFS = Post COVID Functional Scale; hs troponin T = high sensitive Troponin T; NT-proBNP = N-terminal pro brain natriuretic peptide; Anti-RBD = antibody against receptor binding domain / spike protein; LV = left ventricular; LVEF = left ventricular ejection fraction; TAPSE = tricuspid annular plain systolic excursion; LAVI = left atrial volume index; LVEDV index = left ventricular end diastolic volume indexed; RVEDD = right ventricular end diastolic diameter; RAA = right atrial area; sPAP = systolic pulmonary artery pressure.
